# How to Trick Your Opponent: A Review Article on Deceptive Actions in Interactive Sports

**DOI:** 10.3389/fpsyg.2017.00917

**Published:** 2017-05-31

**Authors:** Iris Güldenpenning, Wilfried Kunde, Matthias Weigelt

**Affiliations:** ^1^Department of Sport and Health, University of PaderbornPaderborn, Germany; ^2^Department of Psychology, Würzburg UniversityWürzburg, Germany

**Keywords:** review article, deceptive actions, perception, anticipation, expertise

## Abstract

Performing deceptive actions is a wide-spread phenomenon in sports and it is of considerable practical relevance to know whether or not a fake or a disguised action decreases the opponents’ performance. Therefore, research on deceptive actions for various sport disciplines (e.g., cricket, rugby, martial arts, soccer, and basketball) has been conducted. This research is scattered, both across time and scientific disciplines. Here, we aim to systematically review the empirical work on deceptive actions in interactive sports and want to give an overview about several issues investigated in the last decades. Three main topics of the detected literature were discussed here: (1) the role of expertise for the recognition of deceptive actions, (2) the cognitive mechanisms underlying the processing of deceptive actions, and (3) the pros and cons of *in situ* research designs. None of these themes seems to be settled and therefore, they should be considered in future research agendas.

## Introduction

In many competitive sports, it is a common practice that performers hide information or provide misleading cues about their current intentions regarding their own future actions. Hiding cues is referred to as disguised action (cf. Jackson et al., 2006). A disguised action, for example, is performed by an attacking volleyball player, who tries to hide for as long as possible whether he/she plays a smash or a lob (Güldenpenning et al., 2013). An example of misleading cues are head fakes in basketball, where a player passes to the right side, while simultaneously looking to the left side (Kunde et al., 2011; Weigelt et al., 2016). The core assumption of both types of these deceptive actions is that providing little information or invalid information about one’s own action intention increases the chance of outperforming the opponent (Schmidt and Lee, 2005). In view of the ubiquitous use of deceptive actions in sport practice, it is fair to say that research regarding the efficiency and boundary conditions of such actions has been rather sparse (though increasing recently) and scattered over different fields, such as sport science, cognitive science, and neuroscience. Here, we aim to give an overview over this research and focus on three main topics, which seem to be of special interest for current and future research in the field of sport psychology. Noteworthy, this paper does not include the variety of studies with “illegal” deceptive actions in sports. For example, a soccer player who pretends to be fouled without actually being it (Morris and Lewis, 2010; Pizzera and Raab, 2012; Morgulev et al., 2014), the use of doping to enhance performance (Zurloni et al., 2015), or the financial manipulation of games and competitions. This review only considers studies of legal deceptive behavior in sport.

The first topic (Key topic 1), that is prominent in the literature on deceptive actions, addresses the role of athletic expertise, which is also a classical topic in sport psychology, and more generally, in sports science research (e.g., Ericsson, 2003; Mann et al., 2007). A core issue related to expertise in sports is the question, in how far both visual expertise and motor expertise contribute to experts’ potential superiority in action recognition. Research on action perception and anticipation of non-deceptive actions shows consistently that motor expertise benefits perception and anticipation in sports, especially in interactive game sports and martial arts (Abernethy, 1987; Mann et al., 2007). These superior skills of expert athletes are based on cognitive processes, which rely on a common representation of perception and action (so called perceptual resonance, see Schütz-Bosbach and Prinz, 2007). However, there is also much empirical evidence for the notion that visual expertise based on perceptual experience or deliberate visual-perceptual training also benefits action perception (Hagemann and Memmert, 2006; Abernethy et al., 2012). Therefore, the present review article discusses studies on the relative contribution of visual expertise and motor expertise for the perception of deceptive actions. Within this context, neurophysiological (i.e., fMRI) studies are also considered to illuminate potential motor simulation processes in the brain, when perceiving deceptive actions.

The second topic (Key topic 2) addresses the cognitive mechanism underlying the processing of deceptive actions. This topic predominantly relates to differences between the processing of non-deceptive actions and deceptive actions (i.e., automatic vs. inferential processing, response biases, visual search strategies). Also, the aspect of conflict processing invoked by misleading movement cues (e.g., a head fake) is discussed.

The third topic (Key topic 3), which we consider in the present review article, is a methodological one: Sport scientists have criticized a lack of realistic-like examinations for many years (Janelle and Hillmann, 2003). That is, even though athletes possess both extraordinary perceptual skills and also motor skills, they are typically tested in laboratory settings, which only require the use of perceptual skills, mainly based on processing visual cues (e.g., while anticipating future events in video scenes). The execution of specific motor skills is most often not required, for example, when responses entail only index finger presses. Accordingly, realistic-like examinations on deceptive actions are specifically attended to in a separate section. We think that these topics provide a matrix for the current state of research and will help to identify questions, which have not been considered enough in the past.

## Methods

### Search Strategy

Five electronic databases [*Scopus*, *Web of Science ‘all databases,’ PsycINFO and PsycARTICLES (combined search via EBSCOhost)*, and *SPORTDiscus]* were used for the search of relevant literature. The present review searches for literature published from 1985 up to September, 2016. The English search terms ‘fake’, ‘fool’, ‘feint’, ‘disguise’, ‘body bluff’, and ‘deception,’ were combined with action, sport, and movement. In the *Scopus* and the *SportDiscus* database, the Boolean term ‘fool OR fake OR feint OR disguise OR body bluff OR deception AND movement OR action OR sport’ was applied. For the databases *Web of Science* and *PsycINFO/PsycARTICLES*, search terms were used successively (e.g., first search term: fake AND action; second search term: fake AND sport; third search term: fake AND movement; etc.). The primary literature search resulted in a total of 4580 articles (including duplicates between databases).

### Study Selection

**FIGURE 1:**
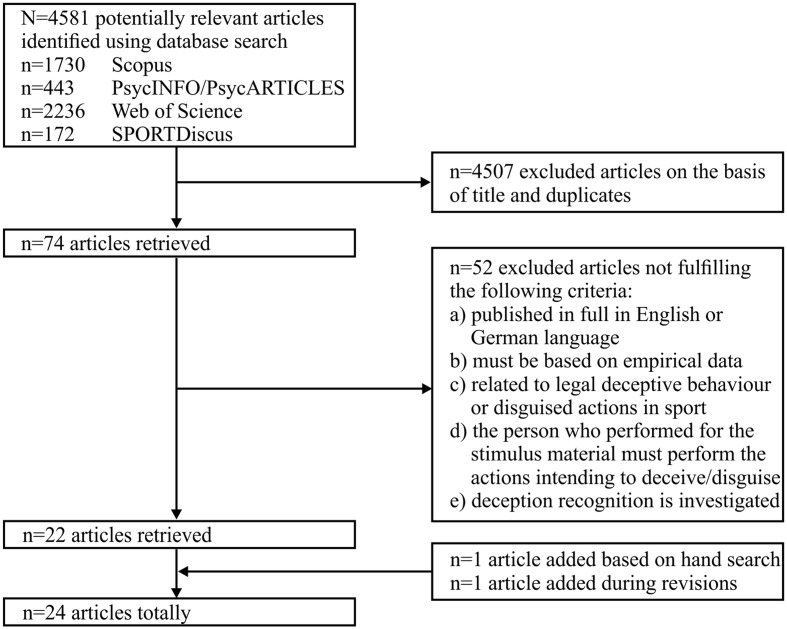
Overview of the search and screening process for the relevant literature.

The search and screening process for relevant literature is shown in **Figure 1**. The first author initially checked the titles of all retrieved studies, removed the duplicates, and excluded those studies, which were obviously related to other fields of research (e.g., lie telling). Article titles not clearly signaling the field of research were not excluded in this step. The initial screening resulted in 74 remaining articles, which were further screened for eligibility based on the following criteria: (a) the study must be published in full in English or German language in a peer-reviewed journal, (b) the study must be based on original data, (c) the study must be related to *legal* deceptive behavior in sports, (d) the person, who performed in the stimulus material, must carry out the actions with the intention to deceive the opponent, (e) the study investigates aspects of deception perception (e.g., excluding research on biomechanical parameters of deceptive actions). Article abstracts and full texts were used to perform a thorough check of these criteria. After this step, 22 articles were identified. The reference sections of each of these 22 articles were then screened by hand, in order to double check for studies, which have been potentially missed out on during the previous steps of the selection process. As a result, one more article was found and added to the list. One further article was added during the revision process (as suggested by one reviewer). An overview of the characteristics of the studies included in the review is provided in a Supplementary Table S1.

## Findings

### General Findings

The 24 articles included in this review involve a total population of 1166 participants (893 male, 273 female), being tested in 67 independent samples. Thirty-seven of the samples contain athletes of different skill level, 30 of the samples contain novice participants and participants with minimal (no competition) experience. Twenty-one studies directly compared anticipation performance between non-deceptive and deceptive actions. Seventeen of these studies indicated that deceptive actions hamper anticipation performance.

### Key Topic 1: The Role of Athletic Expertise for Recognizing Deceptive Actions in Sports

Sixteen studies on different kinds of deceptive actions demonstrated a general superiority in expert athletes compared to novices. These included faking in rugby (Jackson et al., 2006; Brault et al., 2012; Henry et al., 2012; Mori and Shimada, 2013), deceptive soccer moves (Smeeton and Williams, 2012; Bishop et al., 2013; Wright et al., 2013; Wright and Jackson, 2014), faked penalty throws in team handball (Cañal-Bruland and Schmidt, 2009; Cañal-Bruland et al., 2010), fake passes in basketball (Sebanz and Shiffrar, 2009; Weigelt et al., 2016), and disguised actions in tennis (Rowe et al., 2009), beach-volleyball (Güldenpenning et al., 2013), cricket (Adams and Gibson, 1989), and martial arts (Güldenpenning et al., 2015). Notably, there is one study in French boxing, showing that higher skilled boxers produced more false alarms in response to fakes than lower skilled boxers (Ripoll et al., 1995).

Most of these studies revealed that both skilled and unskilled participants were fooled by deceptive actions, but the performance of skilled athletes does not decrease as much as that of novices (Rowe et al., 2009; Brault et al., 2012; Henry et al., 2012; Smeeton and Williams, 2012; Bishop et al., 2013; Mori and Shimada, 2013; Wright and Jackson, 2014). However, the study of Jackson et al. (2006), investigating anticipation performance of attacking movements in rugby, indicated that performance differences between novices (*N* = 14) and expert athletes (*N* = 14) are caused by a decrement in performance in novices for deceptive trials. In contrast, experts performed equally well in non-deceptive and deceptive trials. Cañal-Bruland and Schmidt (2009) also found that novices (*N* = 50), but not skilled team-handball field players (*N* = 50) and skilled team-handball goalkeepers (*N* = 25), performed less well, when judging fake penalty throws in team handball. A further study of Cañal-Bruland et al. (2010) came to similar results.

In view of these studies, the effect of skill level on the recognition of deceptive actions can be summarized as follows: First, skilled athletes perform better than novices, when providing perceptual judgments about non-deceptive and deceptive actions. Thus, the general advantage of expert athletes for using visual information efficiently in advance to better anticipate action outcomes (Mann et al., 2007) seems to generalize to the recognition of deceptive actions. Second, perceiving deceptive actions generally result in a decrement of performance, both in skilled and unskilled participants, but expert athletes seem to be less susceptible to deceptive actions than less skilled athletes or novices (e.g., Brault et al., 2012; Bishop et al., 2013; Wright and Jackson, 2014). Only few studies did not find a decrement in performance in expert athletes to deceptive actions as compared with non-deceptive actions (Jackson et al., 2006; Cañal-Bruland and Schmidt, 2009; Cañal-Bruland et al., 2010). Thus, it seems that deceptive actions generally are an efficient strategy to mislead the opponent, even in professional sport. Studies investigating why experts are less susceptible to deceptive actions than novices are presented below.

#### The Role of Visual and Motor Expertise

Both visual expertise and motor expertise may contribute to better anticipation skills in expert athletes. An explanation for the impact of *motor* expertise is provided by the common-coding theory (Prinz, 1997; Hommel et al., 2001; Schütz-Bosbach and Prinz, 2007). It proposes that the perception and the production of actions share common representations and thus, are intrinsically linked by their common codes. This link is suggested to be bidirectional, meaning that observing an action induces a disposition to execute this action (motor resonance) and producing or even possessing the motor ability to produce an action increases the perceptual sensitivity to similar actions (perceptual resonance, Schütz-Bosbach and Prinz, 2007). Thus, expert athletes, who can rely on better motor representations, have the ability to better perceive (and to better predict) motor actions (and their outcomes) of their own movement repertoire (Calvo-Merino et al., 2006; Casile and Giese, 2006; Aglioti et al., 2008).

Regarding the impact of *visual* expertise, it is argued that the recognition and pick-up of information becomes more efficient through perceptual experiences. More precisely, visual expertise results in optimized attention allocation and cue utilization and, consequently, enables expert athletes to extract more task-relevant information than non-experts (Mann et al., 2007; Huys et al., 2009; Williams et al., 2011). Studies using different experimental manipulations to investigate the relative contribution of visual expertise and motor expertise are discussed in the next sections.

#### Testing Specific Groups

Cañal-Bruland and Schmidt (2009) conducted a study on penalty throwing in team handball, testing participants with different domain-specific knowledge to dissociate the relative influence of visual expertise and of motor expertise. The authors suggested that skilled team-handball field-players (*N* = 50) have similar visual expertise and motor expertise, whereas skilled team-handball goalkeepers (*N* = 25) have much visual expertise, but only small motor expertise (i.e., for the to-be judged penalty throws). Novices (*N* = 50) have neither visual expertise nor motor expertise. Participants’ task was to judge whether video scenes of penalty takers depict a direct throw at the goal (throwing condition) or a fake throw (deception condition). The analysis of the accuracy scores revealed that both team-handball field-players and team-handball goalkeepers were more accurate in their throw/non-throw decisions than novices. There was no difference between the two expert groups. Because team-handball field-players and goalkeepers have equal visual experience, but field players additionally have extensive motor experience, the authors argue that motor expertise does not seem to substantially contribute to the perceptual judgment of the direct throw and fake actions. Rather, visual experience can explain the difference between the skilled groups and the novices.

#### Viewing Perspective

Cañal-Bruland et al. (2010) conducted a further study and manipulated the viewing perspective of the model athlete (i.e., team-handball penalty-taker) shown in the video stimuli. This procedure is a useful strategy to distinguish perceptual and motor expertise (Prasad and Shiffrar, 2009). An impact of motor expertise is suggested to be viewpoint independent, whereas visual expertise strongly depends on the viewpoint. In the study of Cañal-Bruland et al. (2010), team-handball field-players (*N* = 26), team-handball goalkeepers (*N* = 19), and novices (*N* = 20) judged direct throws and fake throws, which were both presented in a front view perspective and a side view perspective. The analysis of the accuracy scores showed that deceptive throws could be better recognized from the front view perspective than from the side view perspective. Importantly, both skilled groups were similarly able to recognize the deceptive actions as fake throws above chance level, and no differences in performance were observed. Hence, the lack of motor expertise in team-handball goalkeepers did not lead to a decrease in their performance, when compared to field players with motor expertise. At the same time, however, this result indicates that the specific visual expertise of goalkeepers for the front view perspective did also not benefit their recognition performance more than those of field players. Accordingly, neither visual expertise nor motor expertise alone can explain the effects of athletic expertise for recognizing deceptive actions.

Similar results were provided by Sebanz and Shiffrar (2009, Experiment 2), who also manipulated the viewing perspective. They presented point-light displays (PLD) of basketball passes and fake passes and found a better performance for the front view perspective than for the side view perspective, both for expert athletes (*N* = 14) and for novices (*N* = 8). It thus seems that sport actions can be generally better identified from a front-view perspective than from a side-view perspective (see alsoTroje et al., 2005), independent of the observers kind of expertise.

#### Visual Training Intervention

Güldenpenning et al. (2013) investigated whether or not visual training would benefit novices’ ability in recognizing a disguised attack hit in beach-volleyball (Experiment 2). A response-priming experiment with photographic stimulus material of the smash and the poke-shot was conducted and applied in a pre-post-test design. That is, between taking part in the reaction time experiment, novice participants (*N* = 16) received two specific video training interventions (for 25 min each). Analyses showed that the visual training did not improve the ability of the participants to recognize the smash shot and the poke shot. Therefore, the authors argued that the superiority in beach-volleyball athletes seems to be rather based on motor expertise than on visual experience.

In contrast to the finding of this study, Alsharji and Wade (2016) could show that national youth and elite team-handball goalkeepers (*N* = 14) increased prediction accuracy for video scenes depicting deceptive and normal penalty throws after seven perceptual training sessions (20 min each). Goalkeepers receiving a placebo video training (*N* = 14) or no training (*N* = 14) did not increase in their judgment performance. Generally, response accuracy was better for direct throws than for deceptive throws in all groups in this study.

Taken the findings on the influences of visual expertise and motor expertise together (Cañal-Bruland and Schmidt, 2009; Sebanz and Shiffrar, 2009; Cañal-Bruland et al., 2010; Güldenpenning et al., 2013), it seems that neither visual expertise nor motor expertise alone can explain the higher level of action recognition in expert athletes. Considering neurophysiological measures during action perception might substantially contribute to an understanding of the mechanisms of experts’ superior anticipation skills, which will be discussed in the following section.

#### Insights from Neurophysiological Measures

It is already known that observing (non-deceptive) actions activates the so called *mirror neuron system* in our brain (Rizzolatti, 2005), which simulates the actions observed (Jeannerod, 2001). Mirroring an observed action is of high relevance for action understanding, action prediction, and also for inferring the intentions of others (e.g., Rizzolatti et al., 2001). The so called *action observation network* (AON; Grafton, 2009) seems to be fundamental for this ability (e.g., Calvo-Merino et al., 2010). The AON consists of different brain areas, including inferior frontal gyrus, dorsal and ventral premotor cortex, and inferior and superior parietal lobule (Zentgraf et al., 2011). Whereas the functional role (i.e., action simulation) of the AON (especially the inferior frontal cortex; IFC) is undisputed for action recognition of non-deceptive actions (e.g., Wright et al., 2010), very little is known about the neural mechanisms of the recognition of deceptive body movements. Presumably, a person observing an action has specific predictions about the kinematics of that action. If, however, a deceptive action is observed, the predicted kinematics is violated (Grèzes et al., 2004). Recognizing that the observed (deceptive) action violates the predicted (non-deceptive) action might modulate the activity in the AON would point out that action simulation, which is based on motor representations, is also critical for recognizing deceptive actions (Tidoni et al., 2013).

Two studies using fMRI measures were conducted on deceptive soccer moves (Bishop et al., 2013; Wright et al., 2013), aiming to investigate the neural responses to normal and deceptive moves in participants with different skill level. In the temporal occlusion study on deceptive soccer moves of Wright et al. (2013), the analysis of the fMRI data for higher-skilled soccer players (*N* = 17), lower-skilled soccer players (*N* = 17), and female novices (*N* = 17) revealed patterns of activation, which are in line with previous fMRI studies in the context of anticipation skill in sport (e.g., Wright et al., 2010, 2011). That is, specifically strong activations in the intraparietal sulcus and the premotor cortex (as part of the AON) were mediated by participants’ level of athletic expertise. Interestingly, additional activations of limbic and subcortical structures point to an involvement of the *social network* (SN; Grafton, 2009), which seems to be a specific aspect of deceptive action observation. Similar results were provided by Bishop et al. (2013), who also observed stronger neural responses in high-skilled observers (*N* = 13) than in intermediates (*N* = 13) and in novices (*N* = 13) in cortical and subcortical structures.

The main findings of these studies were (1) that perceiving deceptive actions activated the AON, comparable to when observing non-deceptive actions, and (2) the AON-activity was more pronounced in highly skilled observers than in less skilled observers. These findings point out that action simulation is also critical for inferring deceptive actions and is strengthened by motor expertise. Activations of the SN might rather point out to an involvement of strategic perceptual processes during deceptive action recognition and are discussed in the concluding section of this article. Behavioral studies investigating cognitive mechanisms of the processing of deceptive actions are presented in the next sections.

### Key Topic 2: Identifying the Cognitive Mechanism of the Processing of Deceptive Actions

#### Processing Mode

Jackson et al. (2006) and Smeeton and Williams (2012) investigated whether non-deceptive and deceptive actions are processed direct perceptually or inferential cue-heuristically. A direct perceptual mode refers to a direct use and pickup of information, whereas an inferential cue-heuristic mode relies on heuristic rules to infer target properties (Runeson et al., 2000; Andersson and Runeson, 2008). Jackson et al. (2006) and Smeeton and Williams (2012) related accuracy rates, revealed in occlusion studies, to the confidence ratings of participants. Accordingly, low accuracy rates with high confidence ratings reflect overconfidence, whereas high accuracy rates with low confidence ratings indicate underconfidence. Cognitive tasks are known to be rated as overconfident, whereas perceptual judgments generally lead to underconfidence. In their temporal occlusion study on deceptive movements, Jackson et al. (2006) showed for a rugby 1:1 situation that confidence ratings were higher for deceptive movements than for non-deceptive movements, both in novices (*N* = 14) and expert athletes (*N* = 14). Also, novices performed worse for deceptive than for non-deceptive actions, whereas skilled athletes performed equally well for non-deceptive and deceptive actions. The authors argue that this result indicated that deceptive actions are recognized based on an inferential mode rather than on a direct perceptual mode.

Smeeton and Williams (2012) accompanied accuracy rates of non-deceptive-exaggerated, non-deceptive, and deceptive soccer penalty kicks with participants’ confidence ratings. Results revealed higher accuracy rates for non-deceptive kicks, compared to deceptive kicks, with confidence ratings not differing between these conditions. In line with Jackson et al. (2006), the authors argue for a cue-heuristic (inferential) processing mode for predicting deceptive actions, due to the overconfidence in the deceptive relative to the non-deceptive kicks. In contrast, non-deceptive actions are processed in the direct perceptual mode. The inferential mode of functioning is argued to be disadvantageous, as attention is drawn to *one* particular movement feature (cf. Jackson et al., 2006). For efficient movement prediction, however, it might be necessary to perceive *multiple* cues simultaneously. Deceptive actions thus might change the way information is being picked up (i.e., from a direct perceptual to an inferential mode), which could explain the decrease in performance. However, the argumentation for different modes of processing can be criticized for the following reason: Participants did not know in advance whether a presented video sequence depicts a non-deceptive or a deceptive action. Therefore, they could not apply different information pick-up strategies. An alternative explanation for the findings of Jackson et al. (2006) and Smeeton and Williams (2012) might be that the confidence ratings were biased by a post-trial impression formation^1^. In the next section, response biases *during* decision making are discussed.

#### Response Bias

Judging an action as being a deceptive action is mostly related to perceptually ambiguous situations. In contrast to novices, experts are familiar with such situations, as they frequently experience them during training and competition. It might also be possible that expertise biases decision making of non-deceptive and deceptive actions (Cañal-Bruland and Schmidt, 2009). Accordingly, Cañal-Bruland and Schmidt (2009) investigated whether or not expertise shifts a persons’ response criterion, such that one response (e.g., fake throw) is preferred over the other (e.g., direct throws). In their team-handball penalty study described above, Cañal-Bruland and Schmidt (2009) applied the signal detection theory (SDT, Green and Swets, 1966), which allows to calculate a response bias. Cañal-Bruland and Schmidt (2009) could show that team-handball goalkeepers (*N* = 25), and field players (*N* = 50) had better discrimination performance between direct throws and fake throws, as compared to novices (*N* = 50), but there was no difference between the skilled groups. The data also indicated that goalkeepers tended to overjudge actions as deceptive. There was no such bias in the novice and the field player group. The authors argue that goalkeepers might favor to judge an action as a deceptive action, because of the expected costs of a missed deception. The response bias in goalkeepers in favor of deceptive actions could result in better keeping rates and might therefore be used strategically by goalkeepers.

Wright et al. (2013) also applied the signal detection measure, but in a soccer study. PLD sequences of a soccer player either approaching and turning with the ball (normal move) or performing a deceptive move (step-over move) before turning were used. The result regarding the response bias was that the higher skilled group (*N* = 17) showed a bias toward identifying moves as being deceptive actions, as compared to a male (*N* = 17) and a female (*N* = 17) group of less skilled players. These results are in line with the findings of Cañal-Bruland and Schmidt (2009), suggesting a strategic component (as reflected in the response bias for deceptive actions) affecting the processing of deceptive actions.

#### Visual Search Strategies

To investigate the perceptual-cognitive mechanisms underlying expert anticipation performance, eye-tracking studies gained growing interest in sport psychology (Savelsbergh et al., 2005). Regarding studies on non-deceptive actions, it has been shown, that experts’ information pick-up is characterized by shorter fixation durations than novices, and by more fixations on task relevant areas than on areas providing redundant information. Also, athletes need less time to first fixate a task relevant area (for overviews, see Mann et al., 2007; Gegenfurtner et al., 2011). Hence, athletic expertise in a certain area appears to change the way how visual information in sports is processed, presumably based on qualitative changes of memory structures (Ericsson and Kintsch, 1995). Mori and Shimada (2013) investigated whether eye movements differ between experts (*N* = 10) and novices (*N* = 10), when anticipating the change of direction of a rugby player, either performed with or without sidesteps (Experiment 1). Results showed that athletes predominantly fixated the hips and the lower trunk of the stimulus model, whereas novices mostly fixated on the chest or upper trunk. The eye movements did not differ between deceptive and non-deceptive movements for both groups. Mori and Shimada (2013) argued that expert athletes fixated areas which can efficiently be used to anticipate the genuine action. In contrast, the areas fixated by novices are supposed to rather convey deceitful information and increase response errors. In this study, expert athletes and novices had equal accuracy rates, but differed in response speed. Fixation areas thus seem to be associated with response speed, but not with response accuracy. The study of Tay et al. (2012) also points to the aspect that visual information pick-up strategies of soccer goalkeepers (*N* = 9) do not differ between deceptive and non-deceptive penalty-kicks.

#### Conflict Processing and Conflict Adaptation

Specific for some deceptive actions is that a faking athlete provides *misleading* information which signals, for example, the contrary direction than the intended passing (e.g., head fake in basketball) or running (e.g., step-over move in soccer) direction. It thus might be of specific importance for an observer to efficiently process the conflict between the deceptive information and the relevant movement information. Conflict processing and mechanisms of conflict adaptation have been paradigmatically investigated for head fakes in basketball (Kunde et al., 2011; Alhaj Ahmad Alaboud et al., 2012, 2016; Weigelt et al., 2016).

Kunde et al. (2011) conducted a study with a series of six reaction-time experiments. Novice participants (*N* = 16) were asked to indicate the passing direction of a basketball player, presented on a (static) picture, by either pressing a left response button (i.e., pass to the left side) or by pressing a right response button (i.e., pass to the right side; Experiment 1). Half of the pictures depicted a basketball player with a head fake, that is, the head orientation was directed opposite to the pass direction. Results showed that responses to the pass direction were slower and less accurate for head fakes, as compared to direct passes (without a head fake). The same authors went on to experimentally explore at which processing stage this so called head-fake effect originates (i.e., perceptual vs. motor stages; Experiment 4, 5, and 6). According to the results of these experiments, the head-fake effect originates at a perceptual level and does not seem to affect motor processing stages.

In another basketball study, Weigelt et al. (2016) investigated whether the perceptual conflict, which is evident in head fakes, is processed differently by basketball experts (*N* = 16), soccer experts (*N* = 24), and non-athletes (*N* = 24). Weigelt et al. (2016) applied the same experimental procedure as Kunde et al. (2011) and showed that the general size of the head-fake effect was not modulated by expertise. However, the head-fake effect in basketball experts disappeared in the current trial *n*, when a head fake was presented in the preceding trial *n*-1. Conversely, the fake effect increased in trial *n*, when a direct pass was presented in the preceding trial *n*-1 (i.e., congruence-sequence effect; CSE). There was no CSE in soccer players and non-athletes. As has been argued, the CSE is an indicator of cognitive control over the processing of task irrelevant information (Gratton et al., 1992; Wühr and Kunde, 2008). Therefore, expert athletes seem to have gained cognitive control over the processing of the gaze direction, at least to some extent.

Processes of conflict control and conflict adaptation can also be investigated with different fake frequency distributions. Alhaj Ahmad Alaboud et al. (2012) used the same approach as Kunde et al. (2011), however, the frequency of fake stimuli was manipulated in a within-subject design. Novice participants (*N* = 24) had to perform three experimental blocks with either 25 or 50 or 75% fake stimuli. The analyses of the response times indicated a head-fake effect for the 25% and the 50% condition, but no head-fake effect for the 75% condition. Further, the head-fake effect was not only modulated by the frequency of the fakes, but also by the preceding target stimulus. The head-fake effect in trial *n* was larger when a target stimulus was preceded by a direct pass (non-fake action) in trial *n*-1. This specific modulation of the head-fake effect based on its fake-frequency distribution is an interesting issue for further research, because the optimal use of deceptive actions during game play is of high practical relevance. In order to transfer these experimental findings to real sport settings, future studies need to be conducted in realistic-like settings, which will be the issue of the next key topic.

### Key Topic 3: The Value of Realistic-Like Examinations

A most recent study by Alhaj Ahmad Alaboud et al. (2016) examined how the experimental set-up modulates the effectiveness of the head-fake effect in basketball. In two different experiments, they tested the effect of response complexity (simple button presses vs. whole body movements; Experiment 1) and of stimulus complexity (static pictures vs. dynamic video scenes; Experiment 2) on the size of the head-fake effect. Quite surprisingly, no effect was found for response complexity in Experiment 1. That is, the size of the head-fake effect was similar for simple button presses and whole body movements (simulating a realistic defensive move in basketball). In Experiment 2, however, results indicated that the size of the head-fake effect substantially increases, when dynamic video scenes are used instead of static pictures (56 ms for video scenes vs. 6 ms for static pictures).

We identified four more studies examining non-deceptive and deceptive actions with (quasi)-realistic settings (Dicks et al., 2010a,b; Brault et al., 2012; Henry et al., 2012). Dicks et al. (2010a) investigated the effect of a fake during penalty kicks on football goalkeepers’ performance in an *in situ* experimental task. Experienced association-football goalkeepers (*N* = 8) had to intercept penalty kicks either performed as deceptive or non-deceptive kicks. The study revealed lower performance (e.g., less saves) to deceptive compared to non-deceptive kicks. Further, deceptive trials led to a greater number of response corrections than non-deceptive trials. Furthermore, goalkeepers’ numbers of saves both for deceptive and non-deceptive trials was better, when only early visual information of the penalty taker was available (i.e., run-up until initiation of the kicking technique). The authors thus argued that goalkeepers should learn to couple their movements to kinematic information provided in the immediate moments before the initiation of the kick, until the foot-ball contact (Dicks et al., 2010a).

Dicks et al. (2010b) conducted another study with football goalkeepers (*N* = 7) which had to intercept penalty kicks either performed as deceptive or non-deceptive kicks. In this study, the authors focused on individual differences of the goalkeepers’ perceptual-motor abilities. The study showed that goalkeeper, who could move faster to one side of the goal, initiated their response to the perceived kicks later than the goalkeeper, who moved slower. The authors argued that the way goalkeeper use information is determined by their action capabilities: Quickly moving goalkeepers can wait longer until movement initiation and have more time to gain information. Thus, they might have a performance advantage above slowly moving goalkeepers.

The study of Brault et al. (2012) revealed comparable results. The authors conducted an experiment and used a virtual reality setting to investigate the detection of deceptive rugby side-steps. Expert rugby players (*N* = 14) and non-rugby players (*N* = 14) watched video scenes of an attacking rugby player presented via a head-mounted display. Participants had to perform a movement to the left/right side, simulating to intercept the attacking rugby player. Analysis of the initiation times revealed that expert athletes waited significantly longer than novices before moving to intercept. Moreover, novices performance to deceptive movements was more error prone than that of expert athletes. It is argued that expert athletes waited longer to get more reliable information about the perceived action. One could also argue that expert athletes have more time to decide, as their physical abilities allow to compensate for a longer decision making process.

Another realistic-like examination has been conducted by Henry et al. (2012), where the effect of a fake on reactive agility performance of higher-skilled (*N* = 14) and less-skilled (*N* = 14) footballers was investigated while performing an agility-test. Participants sprinted toward a screen where an attacking person was presented, who either changed his movement direction directly or faked first and then turned. Participants had to respond to the attacker by turning into the correct direction and running through the appropriate exit gate in a simulated attempt to tackle the attacking person. The analysis of the data showed that the performance similarly decreased for both groups in the deception condition, as compared to the non-deception condition. The decrease of performances occurs both in the decision time (time to initiate a movement as response to the attacker) and in the movement time (time from response initiation to trigger the exit gate). The decrease in performance for fake actions might be both a result of the greater complexity of the cognitive and the motoric component. Interestingly, the influence of fake actions on decision time and movement time was mediated by expertise. The movement time in less skilled football players increased over three times more than in the higher standard football players. Moreover, higher-skilled football players decided earlier for a running direction and needed more time after a fake to correct their decision. This result points out that higher-skilled football players were more susceptible to fakes (cf. Jackson et al., 2006), however, they could compensate the fake-effect with better physical performance. Comparing these results with those of the study of Dicks et al. (2010b) and Brault et al. (2012) described above, expert athletes might possess different strategies for how to quickly react in complex sport scenarios. Based on their physical ability, they could either react very early, risking to fall for a fake (Henry et al., 2012) or they could react later, waiting for more reliable information (Dicks et al., 2010b; Brault et al., 2012).

## Discussion

The first topic, which we address in this article, relates to the role of athletic expertise for recognizing deceptive actions in sports (Key topic 1). The studies presented in this systematic review clearly show that the performance of both expert athletes and novices decreases when they observe deceptive actions, as compared to non-deceptive actions. Expert athletes nevertheless outperform novices in the recognition of deceptive actions and thus, seem to be less susceptible to these actions than novices. The studies detected within the search process contained different experimental manipulations to investigate whether experts’ superiority in detecting deceptive actions is based on visual expertise and/or on motor expertise. One strategy to dissociate visual and motor expertise is to test different groups of participants (Cañal-Bruland and Schmidt, 2009), which are suggested to have both kinds of expertise (e.g., handball field-players), neither kind of expertise (novices), or one kind of expertise (e.g., visual; handball goalkeepers). An alternative approach to investigate visual and motor contributions to deceptive action recognition is to manipulate the perspective of the stimulus material (i.e., front view perspective, side view perspective; Sebanz and Shiffrar, 2009; Cañal-Bruland et al., 2010), as motor expertise is suggested to be viewpoint independent, whereas visual expertise strongly depends on the viewpoint. Last, visual training interventions were conducted to investigate the impact of visual expertise on the ability to detect deception (Güldenpenning et al., 2013; Alsharji and Wade, 2016). From these behavioral studies, the relative role of visual expertise and motor expertise for the recognition of deceptive actions cannot fully be decided upon (see also Makris and Urgesi, 2015). However, two studies using fMRI measures (Bishop et al., 2013; Wright et al., 2013) might provide a more substantial picture: Both studies found specifically strong activations in parts of the AON during the perception of deceptive actions, which was mediated by participants’ expertise. It has been argued that this specific activity in the AON during the observation of deceptive actions is reminiscent of action simulation processes (Tidoni et al., 2013). As simulation processes rely on motoric representations, motor expertise therefore seems to specifically improve the recognition of deceptive actions. Importantly, the involvement of the motor system does not mean that visual experience does not play a role for deceptive action recognition. Instead, optimized attention allocation and cue utilization (Mann et al., 2007; Huys et al., 2009; Williams et al., 2011) could complementarily contribute to deceptive action recognition.

The neurophysiological studies identified here (Bishop et al., 2013; Wright et al., 2013) also provide deeper insights into differences between the processing of deceptive and non-deceptive actions, which directly leads to the discussion of the cognitive mechanism of the processing of deceptive actions (Key topic 2). Specifically, only the perception of fakes activates the so called social network (SN). It is argued that action recognition in the SN is rather based on inferential processes and not purely on simulation processes (Grafton, 2009). Accordingly, not only simulating processes in the AON, but also inferential processes in the SN contribute to fake action recognition. Regarding the role of the AON and the SN, one could also state that the AON is more strongly associated with bottom-up processes, whereas the SN is more strongly associated with top-down processes. As the observation of non-deceptive actions only involves the AON, action recognition might rather be processed directly (i.e., via simulation). As deceptive actions are recognized via the SN, these actions might rather be processed inferentially. These inferential top-down processes might involve visual strategies (and thus visual expertise), for example, an optimized attention allocation to non-deceptive movement features. This suggestion can be supported by the behavioral studies combining judgments about non-deceptive and deceptive actions with confidence ratings (Jackson et al., 2006; Smeeton and Williams, 2012). Together, the behavioral and the neurophysiological studies imply that both visual expertise and motor expertise benefit deceptive action recognition. Certainly, more empirical and theoretical efforts should be undertaken to scrutinize the internal predictive processes of the observer (e.g., Bubic et al., 2010), which might be modulated by bottom-up as well as top-down factors.

In the fMRI study of Wright et al. (2013), also an involvement of limbic and subcortical structures when judging deceptive stimuli was observed. The authors suggest that this activation reflects an affective component of stimuli processing, which is specific for deceptive actions. Emotional responses have also been studied for the observation of non-sports deceptive actions (e.g., box–lifting; Grèzes et al., 2004). Grèzes et al. (2004) conducted an fMRI study and investigated neural events in response to observed actors either trying or not trying to deceive about the real weight of a lifted box. For the judgment of deception, Grèzes et al. (2004) identified an activation network consisting of the amygdala, the anterior cingulate cortex (ACC), the superior temporal sulcus (STS), the orbitofrontal cortex, and the cerebellum. Activations of the amygdala might reflect an emotional response to deceptive stimuli (Dolan, 2002). Activation of the ACC is known to reflect conflict monitoring and the suppression of incorrect response tendencies (Botvinick et al., 1999; Carter and van Veen, 2007). Moreover, Grèzes et al. (2004) argue that the activation of the STS, the orbitofrontal cortex, and the cerebellum are due to a violation of the predicted kinematics when observing a deceptive action. Also, TMS studies showed that recognizing violations of predicted kinematics modulates motor resonance in the observer (Tidoni et al., 2013; Finisguerra et al., 2016). This violation requires an update process of the representation of the mental state of the deceiving person. Together, observing deceptive actions leads to different neural activation than observing non-deceptive actions, as a predicted kinematic is violated and recognized and presumably experienced as a conflict. The recognition of being deceived leads to emotional responses, which might be due to the experience of a potential threat (Grèzes et al., 2006). As it has already been shown that state anxiety and also neuroticism as a personal trait might decrease motor performance (e.g., Barlow et al., 2016), emotional control strategies when responding to deceptive actions might be an interesting issue for further investigations.

Within the topic of the cognitive mechanism of the processing of deceptive actions (Key topic 2), we also detected studies pointing to a response bias for judging actions as deceptive in experts, but not in novices (Cañal-Bruland and Schmidt, 2009; Wright et al., 2013). A comparable result, namely that training and prior experience increased the likelihood of responding deceit as opposed to truth, has also been found for interrogations, implying that mistrust might be a general human tendency (Meissner and Kassin, 2002). Studies investigating visual search strategies (Tay et al., 2012; Mori and Shimada, 2013) and conflict processing (Kunde et al., 2011; Alhaj Ahmad Alaboud et al., 2012, 2016; Weigelt et al., 2016) are discussed with regard to attention as a cognitive mechanism in deception perception. One possibility (besides others) to classify attention processes is to distinguish them as being either intention based (endogenous), or stimulus-driven, automatic (exogenous). Endogenous attention orienting can be regarded as selectively attending to particular target areas. This issue has been extensively investigated in sports science with the eye-tracking methodology (cf. Memmert, 2009). The literature search on deceptive actions in sports resulted in two studies investigating visual search strategies (Tay et al., 2012; Mori and Shimada, 2013). These studies showed that there is no difference in the attended stimulus areas between deceptive and non-deceptive actions. It might be that the attended areas (e.g., certain body parts) comprise information, which can be used to anticipate both the deceptive and the non-deceptive action. Exogenous attention orienting might be of particular relevance for the processing of deceptive actions baring conflicting information, as discussed here for the head fake in basketball (Alhaj Ahmad Alaboud et al., 2012, 2016; Weigelt et al., 2016). It has been argued that the head-fake effect might be based on an automatic processing of the gaze direction. That is, the head-fake effect may be based on attention capture by the irrelevant stimulus feature (i.e., gaze direction) and thus, on a reflexive shift of visual attention to the player’s gaze. Accordingly, the perceptual processing of the relevant stimulus feature (i.e., pass direction) is delayed, because the re-orientation of visual attention from the player’s gaze to the pass takes time. However, distinguishing automatic and intentional processes is challenging here, because gaze direction does not necessarily work completely exogenously. The modulation of the head-fake effect through the preceding trial (congruency-sequence effect; CSE; Gratton et al., 1992) could point to top-down influences of gaze processing. That is, a reduced head-fake effect in trial *n* after a preceding head-fake in trial *n*-1 compared with the head-fake effect after a preceding non-fake trial (Alhaj Ahmad Alaboud et al., 2012, 2016; Weigelt et al., 2016), might reflect expectation-guided preparatory biasing in the anticipation of a forthcoming stimulus. Alternatively, the CSE might reflect an attention set of processing weight in reaction to a processing conflict (for a discussion, see Egner et al., 2010). If the CSE found for the head-fake effect in trial *n* is purely the result of the experience of a processing conflict in the preceding trial *n*-1, the CSE should be quite short-living. This means, that basketball players would only show a reduced head-fake effect if two fakes occur in an immediate sequence (i.e., within 5 s). Investigating the time course of the CSE for the head-fake effect in basketball or comparable conflict situations in other sport settings might be an interesting issue for future research.

In the present review article, the value of realistic-like examinations (Key topic 3) is considered. Therefore, studies resembling realistic perception-action-coupling (Dicks et al., 2010a,b; Brault et al., 2012; Henry et al., 2012) were discussed. It seems that the physical abilities of expert athletes allow for different strategies, when responding to deceptive actions. On the one hand, expert athletes could react very early, risking to fall for a fake, and to correct a wrong response immediately (Henry et al., 2012). On the other hand, expert athletes might react later, waiting for more reliable information, and subsequently perform the motor response quickly (Brault et al., 2012) (also called ‘right-on-time’ hypothesis, cf. Schorer, 2005). Especially, expertise-dependent effects might be modulated by the experimental setting and should be considered when discussing the results of laboratory studies.

An issue, which has been to the best of our knowledge, completely unattended until now, is whether or not the *production* of a fake provokes costs in the performing athlete. Deciding to perform a head-fake in a specific situation instead of performing a direct pass could also result in so called task-switching costs (Kiesel et al., 2010). That is, the performance in task switches (i.e., performing a head-fake) should be worse (i.e., slower reaction times and higher error rates) than the performance in repetitions (i.e., performing direct passes). Importantly, task-switching costs have also been shown to occur when participants decide themselves, which task to perform (Arrington and Logan, 2004, 2005), which generally is the case in sport situations. Moreover, switch-costs decrease if the time to prepare the task increases (e.g., Kiesel and Hoffmann, 2004). Assuming that the preparation for skilled actions is shorter than the preparation for unskilled actions, task-switching costs, and thus fake-action production costs, should be larger for novices than for experts. These rather speculative assumptions are worthwhile to be addressed in future research.

## Author Contributions

All authors listed, have made substantial intellectual contribution to the work, and approved it for publication. IG wrote the first version of the MS. WK and MW revised the MS and performed modifications in the MS.

## Conflict of Interest Statement

The authors declare that the research was conducted in the absence of any commercial or financial relationships that could be construed as a potential conflict of interest.

## Footnotes

1 We thank an anonymous reviewer of a previous version of this manuscript for pointing to this alternative explanation.
