# Supplementary material for: How to Trick Your Opponent: A Review Article on Deceptive Actions in Interactive Sports
Source: Front Psychol. 2017 May 31;8:917. doi: 10.3389/fpsyg.2017.00917 (PMC5449506; doi:10.3389/fpsyg.2017.00917)
Supplement: Supplementary file 1 [file Table_1.pdf]

Supplement A

Table 1. Overview of the studies considered in the review article.

Study	Participant information	Design and Methods	Type of sports	Main findings
Adams and Gibson, 1989	Nine batsmen, (three First Grade batsmen, three batsmen were second and third grade club players, three batsmen were fifth and sixth grade club players)	Mixed design. Batsmen had to indicate with button presses the moment of ball release of an observed real bowler. The bowlers both performed normal bowls and disguised bowls with regard to the moment of ball release.	Cricket	Higher skilled cricket batsmen were better able to predict the moment of ball release in observed bowlers than lower skilled cricket batsmen. Deliberate attempts of the bowlers to disguise the moment of ball release were not successful.
Alhaj Ahmad Alaboud et al. (2016)	Experiment 1) – 2): 1) 24 (5 female) novices. *M* age = 23.04 2) 24 (12 female) novices. *M* age = 22.09.	Within-group design. Reaction-time experiments with target stimuli of a basketball player performing a head fake or playing a pass. 1) Participants had to respond as fast and as accurately as possible to the passing direction of the presented basketball player either with pressing a response button or with performing a whole body movement. 2) Static pictures and video scenes were used as stimulus material.	Basketball	Responses to the passing direction of a presented basketball player were slower and less accurate for fake targets compared to non-fake targets (i.e. fake-effect). The complexity of the response (simple button press vs. whole body movement) did not influence the head-fake effect (Experiment 1). The fake-effect enormously increases if video scenes are used, instead of photographic stimulus material (Experiment 2).
Alhaj Ahmad Alaboud et al. (2012)	Experiment 1) – 2): 1) 24 (19 female) novices. *M* age = 23.38 2) 18 (4 female) novices. *M* age = 25.53.	Within-group design. 1) -2) Reaction-time experiments with target stimuli of a basketball player performing a head fake or playing a pass.	Basketball	1) – 2) Participants' reaction times are slower and more error prone to head fakes compared to passes. This fake-effect decreases if the frequency of presented head fakes increases.
Alsharji and Wade (2016)	21 national youth goalkeepers. Playing experience *M* = 7.33 years, *M* age = 16.81 21 elite goalkeepers. Playing experience *M* = 16 years, M age = 28.67 randomly assigned to a control group, a perceptual training group, and a placebo training group (each *n* = 14; 7 elite + 7 youth)	Mixed design. Perceptual training study to improve anticipation performance in goalkeepers for normal and deceptive penalty throws. The perceptual training group was compared with a group receiving a placebo video training and a standard control group.	Handball	Only the Perceptual training group increased anticipation performance. Both for normal and deceptive throws response accuracy (RA) was better after the perceptual training, however, the increase of the RA was lower for deceptive throws. RA was generally higher for normal than for deceptive throws.
Bishop et al. 2013	11 low-skill anticipators. Competitive experience *M* = 2.4 years. 11 intermed.-skill anticipators. Competitive experience *M* = 10.2 years. 11 high-skill anticipators. Competitive experience *M* = 13.2 years.	Mixed design fMRI; temporal occlusion experiment with video stimuli of a dribbling soccer player performing a stepover in 50% of the trials.	Soccer	Reduced prediction accuracy for early occluded videos and for deceptive actions (i.e. stepover). Higher skilled participants outperformed lower-skilled counterparts under deceptive conditions. Greater activation of cortical and subcortical structures in higher skilled athletes.
Brault, et al., 2012	Experiment 1) – 2): 1) 14 expert rugby players. International playing experience *M* = 13.3 years, *M* age = 23.4 14 non-rugby player. *M* age = 22.6 2) 12 expert rugby players. *M* age = 23.9 12 non-rugby players. *M* age = 22.6.	Mixed design. 1) -2) Head mounted display to create a virtual environment; occluded videos of a virtual attacking rugby player either performing a side-step (fake) or simply running past the defender.	Rugby	1) -2) Expert rugby players outperformed novices in judging deceptive movements. Expert rugby player wait longer than novices before initiating a movement to pick up more relevant information.

Cañal Bruland and Schmidt, 2009	50 skilled handball field players (fourth German division).<br>Playing experience *M* = 15.2 years, *M* age = 26.1.<br>25 skilled handball goalkeepers (fourth German division).<br>Playing experience *M* = 16.9 years, *M* age = 26.1,<br>50 male novices<br>*M* age = 25.9.	Mixed design.<br>Video judgement task with video scenes of penalty taker in handball either performing a shot or a fake.	Handball	Skilled handball players outperformed novices in discriminating shots from fakes. No differences in perceptual sensitivity were found between the goalkeepers and the field players. Only Goalkeepers were significantly biased to judge movements as deceptive.
Cañal Bruland, et al. 2010	26 skilled handball field players (highest Dutch division or national youth team of the Netherlands).<br>Playing experience *M* = 14.4 years, *M* age = 21.2.<br>19 skilled handball goalkeepers (highest Dutch division or national youth team of the Netherlands).<br>Playing experience *M* = 12.3 years, *M* age = 20.3.<br>20 male novices. *M* age = 24.4.	Mixed design.<br>Video judgement task with video scenes of penalty taker in handball either performing a shot or a fake. The videos were presented from the front view or the side view perspective.	Handball	Expert players and goalkeepers outperformed novices in detecting deceptive movements. There were no differences between field players and goalkeepers. Recognition of deceptive actions was more accurate from the goalkeeper's front view than from the side view.
Dicks, Button, & Davids, 2010	8 soccer goalkeepers (at least New Zealand Southern Premier league or equivalent).<br>Playing experience *M* = 11.63 years, *M* age = 22.8.	Within-group design.<br>In-situ examination of goalkeepers' performance to a players' deceptive or non-deceptive penalty kicks. Opaque/translucent goggles were used to temporally manipulate access to information.	Soccer	Goalkeeping performance was better in non-deception trials than in deception trials.
Dicks, Davids, & Button, 2010	7 soccer goalkeepers.<br>Playing experience *M* = 11.7 years, *M* age = 23.4.	Within-group design.<br>In-situ examination of goalkeepers' performance to a players' deceptive or non-deceptive penalty kicks. Individual analyses were calculated.	Soccer	Differences in the action capabilities of goalkeepers affected the timing and accuracy of movement response behaviors. Faster goalkeepers tended to wait until later before initiating movement in comparison with slower goalkeepers.
Güldenpenning, et al. 2015	Experiment 1) – 2)<br>1) 22 athletes of martial arts (7 female).<br>Training experience *M* = 8.4 years, *M* age = 24.4.<br>22 novices (7 female).<br>*M* age = 24.5.<br>2) 22 athletes of martial arts (9 female).<br>Training experience *M* = 9.8 years, *M* age = 25.6.<br>28 novices (15 female).<br>*M* age = 25.3.	Mixed design.<br>1) – 2) Masked priming experiment. Target pictures of a front kick or a roundhouse kick (fake). Were preceded by a not consciously perceivable prime of one of the techniques.	Martial arts	1) – 2) Athletes and novices are not differently sensitive to unconscious perceptual but to unconscious motor priming effects.
Güldenpenning, et al., 2013	Experiment 1) – 2)<br>1) 16 (4 female) skilled beach-volleyball player (A-Cup and B-Cup competitors).<br>Training experience *M* = 9.4 years, *M* age = 23.7.<br>16 (4 female) novices.<br>*M* age = 24.9<br><br>2) 16 (6 female) novices.<br>*M* age = 26.3.	Mixed design.<br>Priming experiment. Participants had to judge whether a presented target picture presents a smash or a poke shot (fake). Before the target, a prime picture of one of the techniques appeared.<br><br>Within-group design<br>Participants took part in the priming experiment twice. Between the tests, a visual training intervention was applied.	Beach-Volleyball	1) Athletes are able to recognize fake and no-fake actions at an earlier point in time than novices.<br>2) This result seems rather be due to athletes' motor and not their perceptual expertise.

Henry, et al. (2012)	14 semi-professional Australian footballers. *M* age = 23.0. 14 amateur Australian footballer. *M* age = 21.0.	Mixed design. Reactive agility test with video stimuli of an attacking faking or no-faking football player.	Football	Agility time was longer for fake trials compared to no-fake trials. Agility time was shorter for higher skilled than for lower skilled football players. Movement time in lower skilled players increased three times more than for higher skilled players in the fake condition.
Jackson, et al. (2006)	14 rugby union players. Playing experience M = 14.4 years, *M* age = 22.3. 14 novices. *M* age = 24.4.	Mixed design. Temporal occlusion experiment with an attacking rugby player either changing direction with or without fake to the right or left side. Participants had to judge direction change. Judgement accuracy and confidence ratings were recorded.	Rugby	Novices were susceptible to deceptive movements whereas rugby players were not. Both groups were more confident on trials containing deceptive movement.
Kunde, et al. (2011)	Experiment 1) – 6): 1) 16 novices (12 female). *M* age = 24.5. 2) 16 novices (13 female) *M* age = 23.0. 3) 32 novices (25 female), *M* age = 23.3. 4) 16 novices (12 female) *M* age = 21.4. 5) 16 novices (14 female) *M* age = 22.9. 6) 16 novices (14 female) *M* age = 24.8.	Within-group design. 1) – 6) A series of Reaction time experiments; Participants had to judge whether a statically presented basketball player, who performed a head fake or not, passes to the right or to the left side. Different manipulations within this basic experiment had been conducted to investigate the source and the origin of the feint effect.	Basketball	1) – 6) Responses to head fakes are slower and more error prone compared to responses to normal passes. This fake-effect is independent of response speed, the practice with the task, and the presence of a fake in the immediately preceding trial. The head direction opposite to the pass direction hampers perceptual encoding of the relevant pass direction and thus originates at a perceptual processing stage.
Mori and Shimada (2013)	Experiment 1) – 3) 1) 10 collegiate rugby players. Playing experience *M* = 9.0 years, *M* age = 23.2. 10 non-players. *M* age = 22.3. 2) 10 collegiate rugby players. Playing experience *M* = 9.2 years, *M* age = 21.4. 10 non-players. *M* age = 22.3 3) 10 collegiate rugby players. Playing experience *M* = 8.8 years, *M* age = 22.2. 10 non-players. *M* age = 22.8.	Mixed design. 1) – 3) Participants had to judge whether a presented video shows an attacking rugby player, either faking or not, changes direction to the right or left side. RTs and eye-movement data was analyzed. The videos were also used as point-light presentations.	Rugby	1) – 3) Rugby players outperformed novices, but were still susceptible to deceptive movements. Rugby players and novices differed in fixation location. Experts fixated rather honest cues whereas novices fixated deceptive cues. Results revealed with the PLDs resembled those of the results of the video-clips.
Ripoll et al. (1995)	6 expert boxers (French National team). *M* age = 27.3. 6 intermediate boxers (competing at the first class level). *M* age = 23.8, 6 novices (more than one year of praxis, no competition). *M* age = 26.3.	Mixed design. Participants had to respond to video clips showing an attack, an opening or a fake by moving a joystick correlated to the expected behavior. No response was required for a fake.	French boxing	Experts produced more false alarms to fakes than intermediate French boxers. Novices produced less false alarms.
Rowe et al. (2009)	18 expert tennis players (2 female). *M* age = 24.67. 62 novices (38 female). *M* age = 22.26	Mixed design. A temporal occlusion experiment was used to investigate anticipation of disguised and non-disguised ground strokes in tennis.	Tennis	Disguised ground strokes decreases anticipation performance in both groups. Experts were some better than novices in anticipating disguised strokes.
Sebanz and Shiffrar (2009)	Experiment 1) -2) 1) 12 basketball experts (3 female). Playing experience *M* = 12.4 years, *M* age = 21.3. 18 novices (8 female).	Mixed design. 1) Participants watched videos and static pictures of a basketball player making a pass or a fake and had to	Basketball	1) Expert basketball player were only better in detecting deception for video stimuli, but not for photographs. 2) Only experts were able to

	M age = 26.0<br>2) 14 basketball experts (4 female).<br>Playing experience *M* = 8.5 years, *M* age = 19.5.<br>8 novices (5 female).<br>*M* age = 20.6.	report whether the player intended to pass or to fake.<br>2) Point light displays of the movements described for Exp. 1 were used, but from front and half-profile view.		detect perception when purely kinematic information was available. Experts performed better in the profile view.
Smeeton and Williams (2012)	13 skilled soccer players (6 female).<br>Playing experience *M* = 13.2 years, *M* age = 24.9.<br>17 novice soccer players (7 female).<br>Playing experience *M* = 0.4 years, *M* age = 25.7.	Mixed design.<br>Temporal occlusion experiment with video clips of non-deceptive, deceptive, and non-deceptive-exaggerated penalty kicks. Judgement accuracy and confidence ratings were recorded.	Soccer	Skilled players were more accurate than less skilled players. Accuracy rate was best for non-deceptive-exaggerated kicks, middle for non-deceptive kicks, and worse for deceptive kicks. Response accuracy increased from early to late occlusion points. Players were over-confident when anticipating deceptive kicks compared to non-deceptive kicks.
Tay et al. (2012)	Nine male skilled soccer goalkeepers.<br>Playing experience *M* = 13.0 years, *M* age = 24.8	Within-group design.<br>Mobile eye tracking system; video clips of faking and non-faking penalty taker were presented to the goalkeepers which wear a mobile eye tracking system. Goalkeepers had to respond to penalty by either moving the right or the left hand	Soccer	Saving rate in goalkeepers was lower for deceptive than for non-deceptive trials. Visual search patterns did not differ between deceptive and non-deceptive trials.
Weigelt et al. (2016)	16 skilled basketball players (4 female).<br>Playing experience *M* = 14.1 years, *M* age = 24.4<br>24 skilled soccer players (5 female).<br>Playing experience *M* = 16.6 years, *M* age = 22.5<br>24 novices (6 female).<br>No specific playing experience, *M* age = 23.5	Mixed design.<br>Reaction time experiment; Participants had to judge whether a statically presented basketball player, who performed a head fake or not, passes to the right or to the left side.	Basketball	The head-fake effect was of similar size in all expertise groups, however, fake experience in the previous trial removed the fake-effect in the subsequent trial in basketball players, but not in soccer players and novices.
Wright and Bishop (2013)	17 higher-skilled soccer players. *M* age = 22.6.<br>17 lower-skilled soccer players. *M* age = 22.1.<br>17 female novices with minimal soccer experience.<br>*M* age = 20.1.	Mixed design.<br>fMRI; Participants task in the scanner was to determine whether point-light video clips of a soccer player approaching with the ball performs a normal or a deceptive move (step-over). In a second experimental block, participants had to predict the direction of the ball of normal and deceptive moves.	Soccer	Higher skilled players showed greater activations in the action observation network (AON) than lower skilled players. Females showed grater activations in the visual cortex. Activation strength with regard to specific areas differed between deception identification and direction identification.
Wright and Jackson (2014)	Experiment 1) – 2)<br>1) 27 higher skilled soccer players. Playing experience *M* = 13.1 years, *M* age = 20.9.<br>20 lower skilled soccer players. Playing experience *M* = 2.8 years, *M* age = 22.6.<br>2) 19 higher skilled soccer players. Playing experience *M* = 12.2 years, *M* age = 23.0.<br>19 lower skilled soccer players. Playing experience *M* = 6.4 years, *M* age = 22.5.	Mixed design.<br>1) Occlusion experiment with videos and point light displays (PLD) of dribbling attacking players either performing normal or deceptive moves. Participants had to predict the ball direction.<br>2) The PLD of experiment 1 were presented. Afterwards, participants had to respond to target letters either presented on the left hand or the right hand side of the participants.	Soccer	1) Response accuracy was higher on video clips than on PLD. Performance on deceptive movements was worse than on normal moves and higher skilled players performed some better.<br>2) Both groups responded faster to targets congruent with the direction of normal turns, and to targets incongruent with the direction of deceptive turns.
